# Poly[[hexa­aqua­(μ_2_-oxalato-κ^4^ *O* ^1^,*O* ^2^:*O* ^1′^,*O* ^2′^)bis­(μ_3_-pyridine-2,4-dicarboxyl­ato-κ^4^ *N*,*O* ^2^:*O* ^2′^:*O* ^4^)dilanthanum(III)] monohydrate]

**DOI:** 10.1107/S160053681104668X

**Published:** 2011-11-09

**Authors:** Fwu Ming Shen, Shie Fu Lush

**Affiliations:** a Department of Biotechnology, Yuanpei University, HsinChu 30015, Taiwan; b Department of General Education Center, Yuanpei University, HsinChu 30015, Taiwan

## Abstract

In the polymeric title compound, {[La_2_(C_7_H_3_NO_4_)_2_(C_2_O_4_)(H_2_O)_6_]·H_2_O}_*n*_, the La^3+^ cation is nine-coordinated in a distorted LaNO_8_ tricapped trigonal–prismatic geometry formed by three pyridinedicarboxylate anions, one oxalate anion and three water mol­ecules. The oxalate anion is located on an inversion center. The oxalate and pyridine­dicarboxyl­ate anions bridge the La^3+^ cations, forming a two-dimensional polymeric complex parallel to (010). Inter­molecular O—H⋯O hydrogen bonding and weak C—H⋯O hydrogen bonding is present in the crystal structure and π–π stacking [centroid–centroid distance = 3.571 (3) Å] is observed between parallel pyridine rings of adjacent mol­ecules. The uncoordinated water molecule shows an occupancy of 0.5.

## Related literature

For related structures, see: Aghabozorg *et al.* (2011 ▶); Li *et al.* (2007 ▶); Wang *et al.* (2009 ▶).
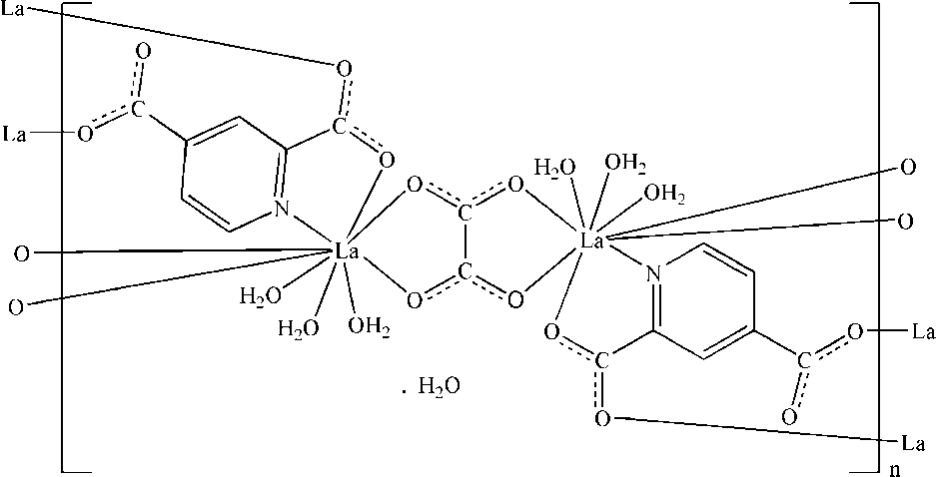


## Experimental

### 

#### Crystal data

[La_2_(C_7_H_3_NO_4_)_2_(C_2_O_4_)(H_2_O)_6_]·H_2_O
*M* *_r_* = 822.16
Triclinic, 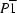

*a* = 6.4614 (8) Å
*b* = 6.6844 (8) Å
*c* = 14.0796 (17) Å
α = 89.735 (2)°
β = 85.266 (2)°
γ = 73.135 (2)°
*V* = 579.85 (12) Å^3^
*Z* = 1
Mo *K*α radiation
μ = 3.73 mm^−1^
*T* = 295 K
0.30 × 0.10 × 0.10 mm

#### Data collection

Bruker SMART 1000 CCD area-detector diffractometer
Absorption correction: multi-scan (*SADABS*; Bruker, 2001 ▶) *T*_min_ = 0.686, *T*_max_ = 0.950
5023 measured reflections
2046 independent reflections
1795 reflections with *I* > 2σ(*I*)
*R*_int_ = 0.035

#### Refinement

*R*[*F* ^2^ > 2σ(*F* ^2^)] = 0.032
*wR*(*F* ^2^) = 0.093
*S* = 1.09
2046 reflections
175 parameters
H-atom parameters constrained
Δρ_max_ = 2.14 e Å^−3^
Δρ_min_ = −2.13 e Å^−3^

### 

Data collection: *SMART* (Bruker, 2007 ▶); cell refinement: *SAINT* (Bruker, 2007 ▶); data reduction: *SAINT*; program(s) used to solve structure: *SHELXTL* (Sheldrick, 2008 ▶); program(s) used to refine structure: *SHELXTL*; molecular graphics: *PLATON* (Spek, 2009 ▶); software used to prepare material for publication: *PLATON*.

## Supplementary Material

Crystal structure: contains datablock(s) global, I. DOI: 10.1107/S160053681104668X/xu5376sup1.cif

Structure factors: contains datablock(s) I. DOI: 10.1107/S160053681104668X/xu5376Isup2.hkl

Additional supplementary materials:  crystallographic information; 3D view; checkCIF report

## Figures and Tables

**Table 1:** Selected bond lengths (Å)

La1—N1	2.726 (4)
La1—O1^i^	2.454 (4)
La1—O3^ii^	2.541 (5)
La1—O4	2.551 (5)
La1—O5	2.543 (4)
La1—O6^iii^	2.550 (5)
La1—O7	2.604 (7)
La1—O8	2.553 (5)
La1—O9	2.612 (7)

Symmetry codes: (i) 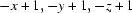; (ii) 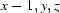; (iii) 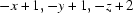.

**Table 2:** Hydrogen-bond geometry (Å, °)

*D*—H⋯*A*	*D*—H	H⋯*A*	*D*⋯*A*	*D*—H⋯*A*
O7—H7*A*⋯O4^ii^	0.84	2.08	2.911 (8)	168
O7—H7*B*⋯O10^iv^	0.83	1.71	2.533 (12)	168
O8—H8*A*⋯O2^v^	0.83	1.83	2.660 (7)	173
O8—H8*B*⋯O6^vi^	0.96	2.03	2.914 (7)	153
O9—H9*A*⋯O6^vi^	0.88	2.27	2.987 (8)	138
O9—H9*B*⋯O10	0.85	1.73	2.390 (14)	133
O10—H10*A*⋯O5^ii^	0.83	2.24	2.885 (12)	135
O10—H10*A*⋯O8^ii^	0.83	2.29	2.924 (15)	133
O10—H10*B*⋯O9^vii^	0.85	1.77	2.591 (17)	163
C5—H5*A*⋯O3^ii^	0.93	2.49	3.164 (7)	130

Symmetry codes: (ii) 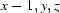; (iv) 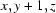; (v) 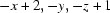; (vi) 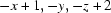; (vii) 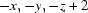.

**Fig. 1.**
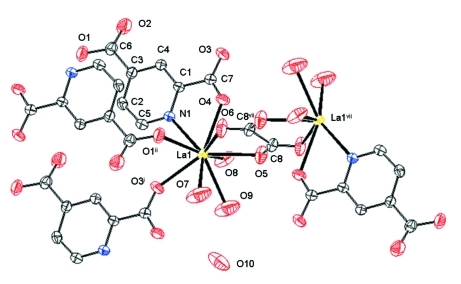
View of the title compound with the atom numbering scheme. Displacement ellipsoids for non-H atoms are drawn at the 50% probability level. All H atoms have been omitted for clarity. [Symmetry code: (i) -1 + x, y, z; (ii) 1 - x, 1 - y, 1 - z; (iv)1+x, y, z.]

**Fig. 2.**
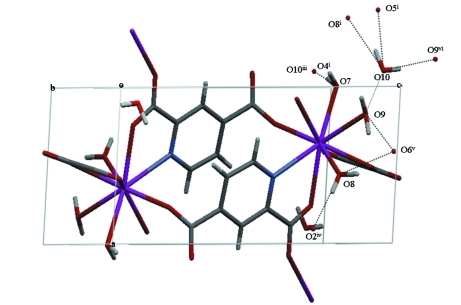
The molecular packing for the title compound. Hydrogen bonds are shown as dashed lines.

**Fig. 3.**
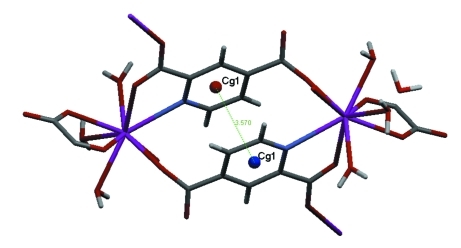
π-π Stacking between pyridine rings [symmetry code: (ii) 1 - x, 1 - y,1 - z.]

## supplementary crystallographic information

### Comment

The pyridine-2,4-dicarboxylic acid (pdcH2) has important coordination functions to metals by either carboxylate bridges between metal centers, to form dimeric complexes or tridentate (O, N, O') chelation to metal ions. Some pydc complexes have been reported (Li *et al.*, 2007; Wang *et al.*, 2009; Aghabozorg *et al.*, 2011).

The symmetric unit of the title compound,{[(LaC_7_H_3_NO_4_)(C_2_O_4_)_0.5_(H_2_O)_3_]_2_.(H_2_O)}_n_, contains two La^III^ atoms, two pyridine-2,4-dicarboxylate(pydc) ligands, one oxalate ligand and six coordinated water molecules. The oxalate ligand are both chelating and bridging, forming an oxalate-bridged dinuclear complex. The La^III^ is nine-coordinated in a distorted tricapped trigonal prismatic geometry by N,*O* atom from a pydc ligand, two O atoms from two pydc ligands, two O atoms from one oxalate ligand and three O atoms from coordinated water molecules (shown as Fig. 1, Table 1). The geometric center of the dimer lies on an inversion center.

The crystal structure contains weak O—H···O and non-classical C—H···O hydrogen bonds. The π-π stacking between two pyridine rings of (pydc) anion fragments with distances of 3.570 (3) Å (1 - *x*, 1 - *y*,1 - *z*) are observed (Fig. 3). The uncoordinated water molecule shows half-occupation.

### Experimental

La(NO_3_)_3_.6H2O (0.1096 g, 0.25 mmole), pydridine-2,4-dicarboxylic acid (0.0418 g, 0.25 mmol) and 4,4'-dipyridine (0.0464 g, 0.25 mmol) were mixed in 10 ml of deionized water. After stirring for 30 min, the mixture was placed in a 23 ml Teflon-lined reactor which was heated under autogenous pressure to 418 K for 48 h and then allowed to cool to room temperature. The brown transparent single crystals were obtained in 41.3% yield (based on La).

### Refinement

The site occupancy factor of the lattice water O10 was refined to 0.509 (16), and was set as 0.5 at the final cycles of refinement. Water H atoms were fixed in chemical sensible positions, thermal parameters were fixed as 0.08 Å^2^. Other H atoms were positioned geometrically with C—H = 0.93 Å (aromatic) and refined using a riding model with *U*_iso_(H) = 1.2*U*_eq_(C).

### Crystal data

**Table:**

[La_2_(C_7_H_3_NO_4_)_2_(C_2_O_4_)(H_2_O)_6_]·H_2_O	*Z* = 1
*M**_r_* = 822.16	*F*(000) = 396
Triclinic, *P*1	*D*_x_ = 2.355 Mg m^−^^3^
Hall symbol: -P 1	Mo *K*α radiation, λ = 0.71073 Å
*a* = 6.4614 (8) Å	Cell parameters from 3390 reflections
*b* = 6.6844 (8) Å	θ = 2.5–25.0°
*c* = 14.0796 (17) Å	µ = 3.73 mm^−^^1^
α = 89.735 (2)°	*T* = 295 K
β = 85.266 (2)°	Columnar, brown
γ = 73.135 (2)°	0.30 × 0.10 × 0.10 mm
*V* = 579.85 (12) Å^3^	

### Data collection

**Table:**

Bruker SMART 1000 CCD area-detector diffractometer	2046 independent reflections
Radiation source: fine-focus sealed tube	1795 reflections with *I* > 2σ(*I*)
graphite	*R*_int_ = 0.035
Detector resolution: 9 pixels mm^-1^	θ_max_ = 25.0°, θ_min_ = 1.5°
φ and ω scans	*h* = −7→7
Absorption correction: multi-scan (*SADABS*; Bruker, 2001)	*k* = −7→7
*T*_min_ = 0.686, *T*_max_ = 0.950	*l* = −16→16
5023 measured reflections	

### Refinement

**Table:**

Refinement on *F*^2^	Primary atom site location: structure-invariant direct methods
Least-squares matrix: full	Secondary atom site location: difference Fourier map
*R*[*F*^2^ > 2σ(*F*^2^)] = 0.032	Hydrogen site location: inferred from neighbouring sites
*wR*(*F*^2^) = 0.093	H-atom parameters constrained
*S* = 1.09	*w* = 1/[σ^2^(*F*_o_^2^) + (0.0639*P*)^2^] where *P* = (*F*_o_^2^ + 2*F*_c_^2^)/3
2046 reflections	(Δ/σ)_max_ = 0.001
175 parameters	Δρ_max_ = 2.14 e Å^−^^3^
0 restraints	Δρ_min_ = −2.13 e Å^−^^3^

### Special details

**Table:**

Geometry. Bond distances, angles *etc*. have been calculated using the rounded fractional coordinates. All su's are estimated from the variances of the (full) variance-covariance matrix. The cell e.s.d.'s are taken into account in the estimation of distances, angles and torsion angles
Refinement. Refinement on *F*^2^ for ALL reflections except those flagged by the user for potential systematic errors. Weighted *R*-factors *wR* and all goodnesses of fit *S* are based on *F*^2^, conventional *R*-factors *R* are based on *F*, with *F* set to zero for negative *F*^2^. The observed criterion of *F*^2^ > σ(*F*^2^) is used only for calculating -*R*-factor-obs *etc*. and is not relevant to the choice of reflections for refinement. *R*-factors based on *F*^2^ are statistically about twice as large as those based on *F*, and *R*-factors based on ALL data will be even larger.

### Fractional atomic coordinates and isotropic or equivalent isotropic displacement parameters (Å^2^)

**Table:**

	*x*	*y*	*z*	*U*_iso_*/*U*_eq_	Occ. (<1)
La1	0.36051 (5)	0.32815 (5)	0.80389 (2)	0.0265 (1)	
O1	0.7674 (8)	0.3427 (7)	0.2806 (3)	0.0392 (16)	
O2	1.0913 (8)	0.2154 (8)	0.3384 (3)	0.0525 (19)	
O3	1.0788 (7)	0.2730 (8)	0.6993 (3)	0.0396 (16)	
O4	0.7592 (7)	0.3177 (9)	0.7799 (3)	0.0521 (18)	
O5	0.5103 (8)	0.2478 (6)	0.9657 (3)	0.0423 (14)	
O6	0.5841 (9)	0.3677 (7)	1.1030 (3)	0.0485 (18)	
O7	−0.0071 (10)	0.5171 (10)	0.8990 (5)	0.0863 (19)	
O8	0.5618 (9)	−0.0608 (7)	0.7835 (4)	0.0542 (19)	
O9	0.1731 (10)	0.0871 (10)	0.9025 (5)	0.0863 (19)	
N1	0.5725 (7)	0.2598 (7)	0.6261 (3)	0.0249 (14)	
C1	0.7769 (9)	0.2729 (9)	0.6137 (4)	0.0257 (17)	
C2	0.5722 (10)	0.2408 (9)	0.4559 (4)	0.0294 (17)	
C3	0.7801 (9)	0.2612 (8)	0.4439 (4)	0.0260 (17)	
C4	0.8843 (9)	0.2739 (9)	0.5250 (4)	0.0285 (17)	
C5	0.4771 (9)	0.2395 (9)	0.5474 (4)	0.0291 (17)	
C6	0.8914 (10)	0.2739 (9)	0.3464 (4)	0.0301 (17)	
C7	0.8804 (10)	0.2893 (10)	0.7040 (4)	0.0341 (19)	
C8	0.5274 (10)	0.3885 (9)	1.0197 (4)	0.0288 (17)	
O10	−0.1015 (18)	−0.0885 (16)	0.9137 (11)	0.072 (5)	0.500
H2A	0.49810	0.22820	0.40340	0.0350*	
H4A	1.02570	0.28300	0.51960	0.0340*	
H5A	0.33850	0.22340	0.55470	0.0350*	
H7A	−0.08470	0.47740	0.86270	0.0800*	
H7B	−0.05250	0.64720	0.89900	0.0800*	
H8A	0.67010	−0.11890	0.74700	0.0800*	
H8B	0.55880	−0.18960	0.81340	0.0800*	
H9A	0.29540	−0.01320	0.89080	0.0800*	
H9B	0.07450	0.05280	0.87580	0.0800*	
H10A	−0.22390	−0.01980	0.89910	0.0800*	0.500
H10B	−0.11240	−0.06870	0.97360	0.0800*	0.500

### Atomic displacement parameters (Å^2^)

**Table:**

	*U*^11^	*U*^22^	*U*^33^	*U*^12^	*U*^13^	*U*^23^
La1	0.0283 (2)	0.0378 (2)	0.0187 (2)	−0.0182 (2)	−0.0020 (1)	0.0042 (1)
O1	0.043 (3)	0.047 (3)	0.025 (2)	−0.009 (2)	−0.004 (2)	0.0078 (19)
O2	0.033 (3)	0.074 (4)	0.039 (3)	−0.001 (2)	0.009 (2)	0.014 (2)
O3	0.028 (2)	0.060 (3)	0.038 (3)	−0.022 (2)	−0.0100 (19)	0.002 (2)
O4	0.037 (3)	0.112 (4)	0.023 (2)	−0.046 (3)	−0.005 (2)	0.007 (2)
O5	0.067 (3)	0.029 (2)	0.032 (2)	−0.013 (2)	−0.015 (2)	0.0013 (19)
O6	0.079 (4)	0.038 (2)	0.036 (3)	−0.022 (2)	−0.030 (3)	0.013 (2)
O7	0.059 (3)	0.072 (3)	0.113 (4)	−0.008 (2)	0.037 (3)	0.026 (3)
O8	0.071 (4)	0.034 (3)	0.053 (3)	−0.016 (2)	0.023 (3)	0.001 (2)
O9	0.059 (3)	0.072 (3)	0.113 (4)	−0.008 (2)	0.037 (3)	0.026 (3)
N1	0.020 (2)	0.029 (2)	0.027 (3)	−0.0093 (19)	−0.0018 (19)	0.002 (2)
C1	0.018 (3)	0.029 (3)	0.031 (3)	−0.008 (2)	−0.003 (2)	0.006 (2)
C2	0.030 (3)	0.037 (3)	0.023 (3)	−0.011 (3)	−0.009 (2)	0.002 (2)
C3	0.025 (3)	0.027 (3)	0.024 (3)	−0.005 (2)	−0.001 (2)	0.004 (2)
C4	0.019 (3)	0.039 (3)	0.027 (3)	−0.008 (2)	−0.002 (2)	0.008 (3)
C5	0.023 (3)	0.036 (3)	0.030 (3)	−0.011 (2)	−0.004 (2)	0.003 (2)
C6	0.033 (3)	0.032 (3)	0.023 (3)	−0.007 (3)	0.001 (2)	0.000 (2)
C7	0.030 (3)	0.046 (4)	0.033 (3)	−0.021 (3)	−0.006 (3)	0.011 (3)
C8	0.032 (3)	0.030 (3)	0.022 (3)	−0.006 (2)	0.000 (2)	0.003 (2)
O10	0.044 (6)	0.044 (6)	0.135 (12)	−0.014 (5)	−0.036 (7)	0.007 (7)

### Geometric parameters (Å, °)

**Table:**

La1—N1	2.726 (4)	O8—H8B	0.9600
La1—O1^i^	2.454 (4)	O9—H9B	0.8500
La1—O3^ii^	2.541 (5)	O9—H9A	0.8800
La1—O4	2.551 (5)	O10—H10A	0.8300
La1—O5	2.543 (4)	O10—H10B	0.8500
La1—O6^iii^	2.550 (5)	N1—C5	1.338 (7)
La1—O7	2.604 (7)	N1—C1	1.346 (8)
La1—O8	2.553 (5)	C1—C4	1.379 (8)
La1—O9	2.612 (7)	C1—C7	1.503 (8)
O1—C6	1.271 (8)	C2—C3	1.386 (9)
O2—C6	1.232 (9)	C2—C5	1.382 (8)
O3—C7	1.251 (8)	C3—C6	1.510 (8)
O4—C7	1.253 (7)	C3—C4	1.388 (8)
O5—C8	1.247 (7)	C8—C8^iii^	1.541 (8)
O6—C8	1.253 (7)	C2—H2A	0.9300
O7—H7B	0.8300	C4—H4A	0.9300
O7—H7A	0.8400	C5—H5A	0.9300
O8—H8A	0.8300		
			
O4—La1—O5	73.91 (15)	La1—O5—C8	121.8 (4)
O4—La1—O7	143.20 (19)	La1^iii^—O6—C8	121.6 (4)
O4—La1—O8	76.08 (19)	La1—O7—H7B	119.00
O4—La1—O9	130.46 (19)	H7A—O7—H7B	105.00
O4—La1—N1	60.09 (14)	La1—O7—H7A	96.00
O3^ii^—La1—O4	136.05 (14)	La1—O8—H8A	129.00
O1^i^—La1—O4	94.15 (17)	H8A—O8—H8B	93.00
O4—La1—O6^iii^	71.03 (17)	La1—O8—H8B	138.00
O5—La1—O7	85.94 (19)	La1—O9—H9A	87.00
O5—La1—O8	78.78 (15)	H9A—O9—H9B	108.00
O5—La1—O9	68.31 (19)	La1—O9—H9B	116.00
O5—La1—N1	129.55 (15)	H10A—O10—H10B	102.00
O3^ii^—La1—O5	143.58 (15)	La1—N1—C5	123.7 (4)
O1^i^—La1—O5	131.93 (14)	La1—N1—C1	118.9 (3)
O5—La1—O6^iii^	62.98 (13)	C1—N1—C5	116.8 (5)
O7—La1—O8	130.39 (19)	N1—C1—C4	122.9 (5)
O7—La1—O9	64.2 (2)	N1—C1—C7	115.1 (5)
O7—La1—N1	143.97 (18)	C4—C1—C7	121.9 (6)
O3^ii^—La1—O7	76.45 (18)	C3—C2—C5	118.7 (5)
O1^i^—La1—O7	76.62 (19)	C2—C3—C6	122.0 (5)
O6^iii^—La1—O7	72.42 (19)	C4—C3—C6	120.1 (5)
O8—La1—O9	66.27 (19)	C2—C3—C4	117.9 (5)
O8—La1—N1	71.47 (16)	C1—C4—C3	119.6 (6)
O3^ii^—La1—O8	88.67 (17)	N1—C5—C2	123.9 (6)
O1^i^—La1—O8	144.45 (16)	O1—C6—O2	126.8 (6)
O6^iii^—La1—O8	134.78 (18)	O2—C6—C3	117.2 (5)
O9—La1—N1	128.72 (18)	O1—C6—C3	116.1 (6)
O3^ii^—La1—O9	75.33 (18)	O4—C7—C1	116.7 (6)
O1^i^—La1—O9	135.03 (19)	O3—C7—C1	119.0 (5)
O6^iii^—La1—O9	115.40 (18)	O3—C7—O4	124.3 (6)
O3^ii^—La1—N1	76.02 (14)	O5—C8—O6	126.8 (5)
O1^i^—La1—N1	74.06 (14)	O5—C8—C8^iii^	116.9 (5)
O6^iii^—La1—N1	114.76 (15)	O6—C8—C8^iii^	116.4 (5)
O1^i^—La1—O3^ii^	74.77 (16)	C5—C2—H2A	121.00
O3^ii^—La1—O6^iii^	136.50 (17)	C3—C2—H2A	121.00
O1^i^—La1—O6^iii^	69.06 (15)	C1—C4—H4A	120.00
La1^i^—O1—C6	137.1 (4)	C3—C4—H4A	120.00
La1^iv^—O3—C7	139.6 (4)	N1—C5—H5A	118.00
La1—O4—C7	128.4 (4)	C2—C5—H5A	118.00
			
O5—La1—O4—C7	158.8 (6)	N1—La1—O1^i^—C6^i^	73.1 (6)
O7—La1—O4—C7	−141.7 (5)	O4—La1—O6^iii^—C8^iii^	−86.9 (5)
O8—La1—O4—C7	76.7 (6)	O5—La1—O6^iii^—C8^iii^	−5.7 (5)
O9—La1—O4—C7	117.6 (6)	O7—La1—O6^iii^—C8^iii^	88.8 (5)
N1—La1—O4—C7	0.3 (5)	O8—La1—O6^iii^—C8^iii^	−41.2 (6)
O3^ii^—La1—O4—C7	3.7 (7)	O9—La1—O6^iii^—C8^iii^	39.8 (6)
O1^i^—La1—O4—C7	−68.6 (6)	N1—La1—O6^iii^—C8^iii^	−129.2 (5)
O6^iii^—La1—O4—C7	−134.8 (6)	La1^i^—O1—C6—C3	102.3 (6)
O4—La1—O5—C8	82.1 (5)	La1^i^—O1—C6—O2	−78.5 (8)
O7—La1—O5—C8	−66.7 (5)	La1^iv^—O3—C7—O4	−11.4 (11)
O8—La1—O5—C8	160.7 (5)	La1^iv^—O3—C7—C1	169.0 (4)
O9—La1—O5—C8	−130.5 (5)	La1—O4—C7—C1	4.4 (9)
N1—La1—O5—C8	106.5 (5)	La1—O4—C7—O3	−175.2 (5)
O3^ii^—La1—O5—C8	−127.3 (5)	La1—O5—C8—O6	174.5 (5)
O1^i^—La1—O5—C8	1.2 (6)	La1—O5—C8—C8^iii^	−5.3 (8)
O6^iii^—La1—O5—C8	5.6 (5)	La1^iii^—O6—C8—O5	174.7 (5)
O4—La1—N1—C1	−5.7 (4)	La1^iii^—O6—C8—C8^iii^	−5.5 (8)
O4—La1—N1—C5	−177.0 (5)	C5—N1—C1—C4	2.4 (8)
O5—La1—N1—C1	−32.9 (5)	La1—N1—C1—C7	9.8 (6)
O5—La1—N1—C5	155.9 (4)	C1—N1—C5—C2	−3.0 (8)
O7—La1—N1—C1	135.5 (4)	C5—N1—C1—C7	−178.3 (5)
O7—La1—N1—C5	−35.7 (6)	La1—N1—C5—C2	168.4 (4)
O8—La1—N1—C1	−90.0 (4)	La1—N1—C1—C4	−169.4 (4)
O8—La1—N1—C5	98.8 (4)	N1—C1—C7—O3	170.2 (6)
O9—La1—N1—C1	−125.6 (4)	N1—C1—C7—O4	−9.4 (8)
O9—La1—N1—C5	63.2 (5)	C4—C1—C7—O3	−10.5 (9)
O3^ii^—La1—N1—C1	176.7 (4)	C4—C1—C7—O4	169.9 (6)
O3^ii^—La1—N1—C5	5.4 (4)	N1—C1—C4—C3	0.1 (9)
O1^i^—La1—N1—C1	98.8 (4)	C7—C1—C4—C3	−179.1 (5)
O1^i^—La1—N1—C5	−72.4 (4)	C5—C2—C3—C4	1.6 (8)
O6^iii^—La1—N1—C1	41.6 (4)	C3—C2—C5—N1	1.0 (9)
O6^iii^—La1—N1—C5	−129.7 (4)	C5—C2—C3—C6	−177.2 (5)
O4—La1—O3^ii^—C7^ii^	−173.4 (6)	C2—C3—C6—O1	25.5 (8)
O5—La1—O3^ii^—C7^ii^	49.4 (8)	C6—C3—C4—C1	176.7 (5)
O7—La1—O3^ii^—C7^ii^	−13.9 (7)	C4—C3—C6—O2	27.4 (8)
O8—La1—O3^ii^—C7^ii^	118.3 (7)	C2—C3—C6—O2	−153.9 (6)
O9—La1—O3^ii^—C7^ii^	52.5 (7)	C4—C3—C6—O1	−153.3 (5)
N1—La1—O3^ii^—C7^ii^	−170.5 (7)	C2—C3—C4—C1	−2.1 (8)
O4—La1—O1^i^—C6^i^	130.4 (6)	O5—C8—C8^iii^—O5^iii^	−180.0 (6)
O5—La1—O1^i^—C6^i^	−157.6 (6)	O5—C8—C8^iii^—O6^iii^	−0.2 (9)
O7—La1—O1^i^—C6^i^	−85.8 (6)	O6—C8—C8^iii^—O5^iii^	0.2 (9)
O8—La1—O1^i^—C6^i^	58.6 (7)	O6—C8—C8^iii^—O6^iii^	180.0 (6)
O9—La1—O1^i^—C6^i^	−56.3 (7)		

Symmetry codes: (i) −*x*+1, −*y*+1, −*z*+1; (ii) *x*−1, *y*, *z*; (iii) −*x*+1, −*y*+1, −*z*+2; (iv) *x*+1, *y*, *z*.

### Hydrogen-bond geometry (Å, °)

**Table:**

*D*—H···*A*	*D*—H	H···*A*	*D*···*A*	*D*—H···*A*
O7—H7A···O4^ii^	0.84	2.08	2.911 (8)	168
O7—H7B···O10^v^	0.83	1.71	2.533 (12)	168
O8—H8A···O2^vi^	0.83	1.83	2.660 (7)	173
O8—H8B···O6^vii^	0.96	2.03	2.914 (7)	153
O9—H9A···O6^vii^	0.88	2.27	2.987 (8)	138
O9—H9B···O10	0.85	1.73	2.390 (14)	133
O10—H10A···O5^ii^	0.83	2.24	2.885 (12)	135
O10—H10A···O8^ii^	0.83	2.29	2.924 (15)	133
O10—H10B···O9^viii^	0.85	1.77	2.591 (17)	163
C5—H5A···O3^ii^	0.93	2.49	3.164 (7)	130

Symmetry codes: (ii) *x*−1, *y*, *z*; (v) *x*, *y*+1, *z*; (vi) −*x*+2, −*y*, −*z*+1; (vii) −*x*+1, −*y*, −*z*+2; (viii) −*x*, −*y*, −*z*+2.
